# 510. Bacteriophages as Drug Delivery Vehicles for Polymyxin B

**DOI:** 10.1093/ofid/ofae631.162

**Published:** 2025-01-29

**Authors:** Yanxi Yang, Irene Chen

**Affiliations:** UCLA, Los Angeles, CA; UCLA, Los Angeles, CA

## Abstract

**Background:**

The goal of this project is to develop a safer, more effective formulation of polymyxin B for *E. coli* bacteremia. Polymyxins are a family of cyclic lipopeptides that display exceptional antibiotic activities toward gram-negative pathogens such as *E. coli*, *Pseudomonas aeruginosa*, and *Acinetobacter baumannii*. The PMB label includes an FDA black box warning for serious nephrotoxicity and neurotoxicity. However, today, with the growing emergence of “superbugs” and a dry antibiotic discovery pipeline, PMB is increasingly used as a last-line therapy to treat systemic infections. Thus, although PMB is a potent antibacterial agent, its toxicity currently prevents its use as an earlier or more widespread treatment.

Transmission electron microscopy (TEM; negative stain) images of anti-LPS phage bound to gram-negative organisms.

**Figure:**
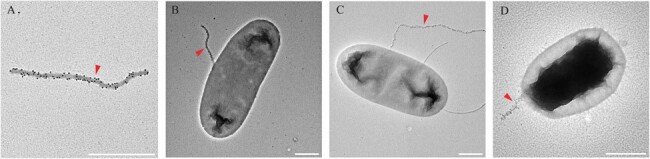

Phages were labeled with gold nanoparticles (dark spheres) coated with antibodies against pVIII, allowing phages to be easily identified (red darts). Gold-labeled anti-LPS phage are shown (A) without bacteria; or incubated with (B) E. coli DH5α; (C) E. coli ATCC BAA 1161; (D) A. baumannii ATCC 19606. Phage width is increased by the labeling reagents. Scale bars = 500 nm.

**Methods:**

We engineered M13 to bind the core antigen of lipopolysaccharide (LPS), enabling binding to a variety of top-priority pathogenic gram-negative bacterial species, including *E. coli, P. aeruginosa, A. baumannii, Klebsiella pneumoniae,* and *Burkholderia cepacia*. Since the anti-LPS phages are able to bind but do not infect (nonviable), they are not cytotoxic by themselves. However, delivery of PMB by the anti-LPS phage confers antibiotic activity. Thus, in our strategy, PMB molecules are conjugated to the anti-LPS phage capsid, and delivered specifically to gram-negative bacterial cells through the specificity and high affinity of the anti-LPS binding domain. We have shown that the PMB – anti-LPS phage conjugate (termed “PMB–Phage” hereafter) effectively lowers the minimal inhibitory concentration of PMB by multiple orders of magnitude *in vitro* compared to PMB sulfate.

MIC and MBC of PMB and PMB-Phage, determined in vitro for several gram-negative organisms.

**Figure:**
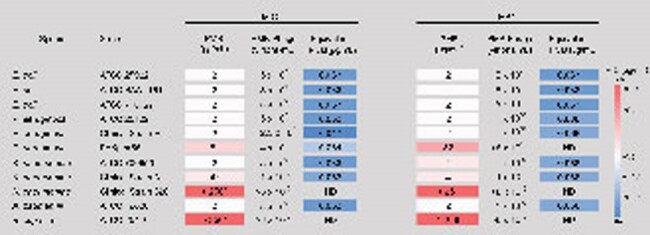

The heat map corresponds to the MIC or MBC values. Equivalent PMB concentrations for PMB-Phage MIC and MBC were calculated from the measured phage concentration and the average amount of PMB loaded per virion. * = resistant. ND = not determined due to technical limitation on phage concentration.

**Results:**

We have already demonstrated following results.

1. A broad-spectrum anti-LPS phage was engineered and verified to bind to several gram-negative bacterial species including *E. coli*.
2. Polymyxin B was conjugated to the anti-LPS phage, creating PMB-Phage, which was highly antibacterial *in vitro*.
3. PMB-Phage is non-toxic to mammalian cells *in vitro* and non-toxic in mice.
4. PMB-Phage is distributed to various organs within 1 hour and has an elimination half-life of approximately 1 day.
5. PMB-Phage appears to be effective in treating *E. coli* bacteremia in a mouse model.

PMB-Phage Biodistribution

**Figure:**
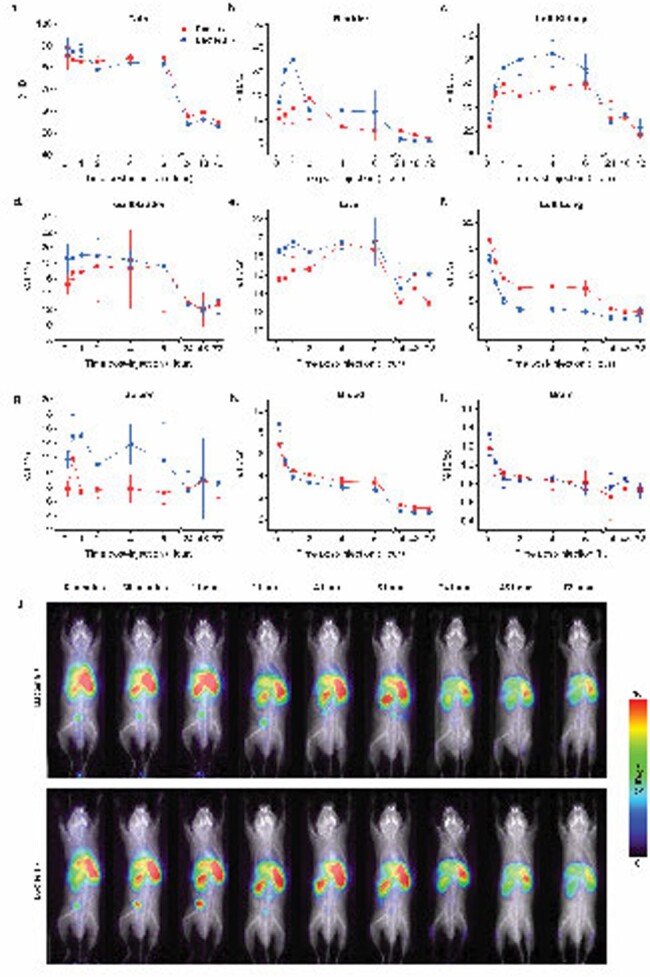

Figure 9: Time course biodistribution of PMB-Phage in various organs, over time in male mice with (red) or without (blue) 1×10^7cfu (non-lethal dose) of E. coli ATCC BAA 1161 injected (n=3). Signals are measured as a percentage of the injected dose (ID) per volume (per cc). Representative biodistribution images (j) of 1 animal.

**Conclusion:**

PMB-Phage will greatly reduce the nephrotoxicity of PMB, enabling safe treatment for a broader patient population.

Liver and kidney blood biomarkers at end point of 7-day toxicity studies.

**Figure:**
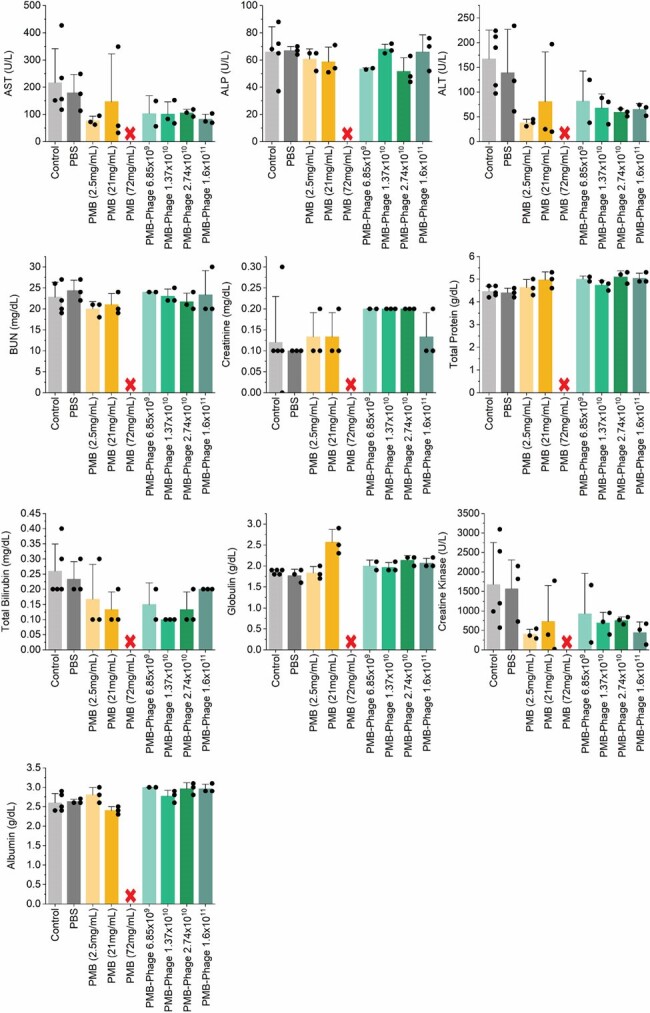

Animals in experimental groups were injected 100µL of test materials through IV tail vein injection daily for 7 consecutive days. Experimental group animals for each condition (N=3), receiving either PMB or PMB-Phage at different doses show no significant difference comparing to control groups of animals group receiving no treatment (N=5) and 1xPBS buffer (N=3), in light and dark grey columns respectively. The only exception being all animals receiving 72 mg/kg body weight PMB (N=3) did not survive a single dose and died on day 1 (indicated by red cross in figure).

**Disclosures:**

**All Authors**: No reported disclosures

